# Clinical Evaluation of Three Direct Pulp Capping Materials in Caries‐Induced Pulpitis of Mature Permanent Teeth: A Randomized Controlled Trial

**DOI:** 10.1002/cre2.70367

**Published:** 2026-05-26

**Authors:** Yutong Li, Kangrui Zeng, Boshu Chen, Qiongyi Kang, Lili Huang, Hong Huang, Tingting Li, Yueping Pan, Shujun Ran, Wenwei Xia

**Affiliations:** ^1^ Department of Endodontics and Operative Dentistry, Shanghai Ninth People's Hospital, Shanghai Jiao Tong University School of Medicine, College of Stomatology Shanghai Jiao Tong University Shanghai China; ^2^ National Clinical Research Center for Oral Diseases, Shanghai Key Laboratory of Stomatology & Shanghai Research Institute of Stomatology Shanghai Jiao Tong University School of Medicine Shanghai China; ^3^ Department of Epidemiology, School of Public Health University of Michigan Ann Arbor Michigan USA

**Keywords:** calcium hydroxide, direct pulp capping, iRoot BP Plus, mineral trioxide aggregate, reversible pulpitis

## Abstract

**Objectives:**

Direct pulp capping (DPC) preserves pulp vitality in caries‐exposed permanent teeth, but clinical evidence comparing different capping materials and identifying prognostic factors remains limited. This study evaluated the success of DPC with iRoot BP Plus, mineral trioxide aggregate (MTA), and calcium hydroxide (CH), and explored factors associated with treatment outcomes.

**Material and Methods:**

In this single‐blind, parallel‐group, superiority randomized trial, 120 patients with caries‐induced reversible pulpitis were equally assigned to CH, MTA, or iRoot BP Plus. The primary outcome was 12‐month success, defined by combined clinical and radiographic criteria. Associations between baseline clinical factors and 12‐month success were evaluated. Secondary analyses included success at 3 and 6 months.

**Results:**

At 12 months, 81 of 120 randomized patients (67.5%) were available for evaluation. The overall success rate was 88.9%. Success rates were 88.5% for iRoot BP Plus (*n* = 26), 93.1% for MTA (*n* = 29), and 84.6% for CH (*n* = 26), with no significant differences between groups (*p* = 0.6). Failures were significantly associated with lesions confined to the proximal surface (*p* = 0.001) at 12 months, while exposure size > 1 mm (*p* = 0.024) and anterior teeth (*p* = 0.018) were additional risk factors at 6 months. Kaplan–Meier analysis showed high survival across all groups (log‐rank *p* = 0.66) and confirmed the influence of these risk factors. No adverse events were reported.

**Conclusions:**

DPC achieved high short‐term success in mature permanent teeth in MTA, CH, and iRoot BP Plus. Prognostic factors such as caries location, tooth position, and exposure size remain critical for case selection.

**Trial Registration:** number: ChiCTR2100047588; Chinese Clinical Trial Register.

## 1 Introduction

Vital pulp therapy (VPT), including direct and indirect pulp capping as well as partial and full pulpotomy, aims to preserve pulp vitality in cases of deep caries approaching or involving the pulp, or when pulp exposure occurs due to trauma or mechanical causes (American Association of Endodontists 2020). Direct pulp capping (DPC) refers to the direct application of a pulp‐capping material onto exposed pulp tissue to stimulate reparative dentin formation and promote pulp healing, thereby maintaining pulp vitality (Islam et al. 2023). The choice of pulp‐capping material is one of the most important determinants of treatment success (Leong and Yap 2021). An ideal capping agent should demonstrate long‐lasting antimicrobial activity, effective sealing ability, biocompatibility, regenerative potential, and ease of clinical application (Giraud et al. 2019).

Calcium hydroxide (CH) and mineral trioxide aggregate (MTA) are the most commonly used materials in current clinical practice. CH is easy to manipulate and, due to its high alkalinity, provides sustained antibacterial action while stimulating reparative dentin formation (Mohammadi and Dummer 2011). However, long‐term studies have shown that CH gradually dissolves, creating pathways for microleakage (Nair et al. 2008) and leading to reduced long‐term success rates (Barthel et al. 2000). MTA, a bioactive endodontic cement, has been shown to provide superior sealing, antibacterial activity, biocompatibility, and pulp‐inductive capacity (Li et al. 2015). It has achieved favorable clinical outcomes in DPC (Duarte et al. 2003), but its widespread use is hindered by drawbacks such as high cost, technique sensitivity, long setting time, and potential for tooth or gingival discoloration (Chen et al. 2018; Fan et al. 2018). Therefore, there is a clear need for novel pulp capping materials that balance biological efficacy with better handling and esthetic properties.

iRoot BP Plus is a premixed, ready‐to‐use, calcium silicate–based bioceramic material with excellent biocompatibility, bioactivity, and favorable handling characteristics (Zheng et al. 2024). Unlike MTA, it does not cause tooth discoloration and is easier to manipulate clinically. Previous in vitro and animal studies have demonstrated that iRoot BP Plus has comparable efficacy to MTA in pulp capping (Shi et al. 2016), with both showing strong apatite‐forming ability. Furthermore, iRoot BP Plus has been shown to promote greater proliferation and adhesion of human dental pulp stem cells compared to MTA (Zhu et al. 2014). However, long‐term clinical data evaluating its performance in mature permanent teeth with caries‐related pulp exposures remain scarce.

In addition to the choice of capping material, other clinical factors—such as patient age, size of pulp exposure, use of rubber dam isolation, and duration of preoperative pain—may also influence DPC outcomes (Miles et al. 2010; Çalışkan and Güneri 2017; Islam et al. 2023; Ricucci et al. 2023). Nonetheless, controversies remain regarding the predictive value of these factors (Prasertsuksom et al. 2024). A clearer understanding of prognostic indicators would enable clinicians to better predict treatment outcomes, improve patient selection, and refine treatment strategies.

Based on this background, the objective of this single‐blind, randomized controlled clinical trial was to evaluate the success rates of DPC using CH, MTA, and iRoot BP Plus in mature permanent teeth with caries‐induced reversible pulpitis. In addition, this study sought to investigate potential prognostic factors influencing treatment outcomes, with the goal of providing evidence to support standardized vital pulp preservation strategies in caries‐induced pulpitis.

## 2 Methods

### 2.1 Participants

This study was conducted in the Department of Endodontics at Shanghai Ninth People's Hospital, Shanghai Jiao Tong University School of Medicine, China. The study was approved by the Institutional Ethics Committee (Ethical number: SH9H‐202‐T418‐3) and was registered in the Chinese Trials Registry (No. ChiCTR2100047588) (Clinical study of vital pulp preservation treatment of carious teeth with pulpitis—ChiCTR2100047588,2100047588 2022). Informed consent was obtained from all the patients prior to treatment. Patients who had deep carious teeth with possible pulp exposure and visited the Department of Endodontics from July 2021 to July 2024 were recruited for the study.

The inclusion criteria were as follows: (1) patients aged ≥ 18 years who provided informed consent; (2) mature permanent teeth with fully developed roots teeth with deep caries extending beyond two‐thirds of the dentin thickness without spontaneous pain; (3) teeth experiencing discomfort when a stimulus such as cold or sweet is applied and goes away within a couple of seconds following the removal of the stimulus; (4) no significant radiographic changes in the periapical region of the suspect tooth, with no evidence of root fracture, resorption, or pulp chamber calcification; (5) teeth showed normal mobility, no swelling or sinus tract on gingiva; (6) Pulp exposure occurring after caries removal.

Teeth were excluded if they were non‐restorable, exhibited vertical root fracture, root resorption, pulp necrosis, or periapical lesions. Cases with moderate to severe periodontitis or furcation involvement, as well as patients who were pregnant, systemically or mentally compromised, or unable or unwilling to cooperate, were also excluded. Teeth were also excluded if hemostasis could not be achieved within 5 min during the procedure.

### 2.2 Study Design

This investigation was a single‐center, parallel‐group, superiority randomized controlled trial. Baseline data, including age, gender, and history of systemic diseases and a history of the chief complaint (including precipitating factors and types of pain) were recorded prior to intervention. A clinical examination was conducted to record the position of the affected tooth, carious tooth surfaces, tooth mobility, and the responses to cold stimuli (responsive (+), exaggerated response (++), or non‐responsive (−) and results of electric pulp testing (EPT). Periapical radiographs were taken to identify the periapical index (PAI) score (Huumonen and Ørstavik 2002).

All direct pulp capping procedures were performed by a single board‐certified endodontist (WX) with more than 10 years of clinical experience in vital pulp therapy, ensuring procedural consistency. Outcome assessments were conducted independently by two calibrated endodontists who were not involved in treatment delivery.

Participants were randomly assigned in equal proportions to one of three treatment groups: MTA, CH, or iRoot BP Plus. Based on prior literature, the expected success rates were 98% for iRoot BP Plus, 86% for MTA, and 65% for CH (Liu et al. 2020; Cushley et al. 2021). Assuming a power of 90% and a significance level of 0.05, the calculated sample size was 32 participants per group. To account for an anticipated 20% dropout rate, the final target enrollment was set at 40 participants in each treatment group.

A random number sequence was generated by a professional independent third‐party institution via SPSS 23.0, which was assigned sequentially to the patients fulfilling the inclusion criteria. The trial was conducted using a single‐blind design. Participants and outcome assessors were blinded to treatment allocation, whereas the operator performing the procedure was not. Unblinding was permitted only in cases of treatment failure or when knowledge of the assigned material was clinically necessary for patient safety and care.

### 2.3 Procedures

After enrollment of the affected tooth, the following procedures were conducted. The tooth was anesthetized using 4% Articaine with epinephrine 1/100,000 (Primacaine; Acteon Pharma, Bordeaux, France) following disinfection with a povidone‐iodine‐soaked cotton swab. Caries was excavated using a high‐speed handpiece with sterile distilled water as a coolant under rubber dam isolation. To ensure optimal visibility, caries excavation was performed under a dental operating microscope (OMS2350, Zumax, Suzhou, China) throughout the entire procedure. If pulp exposure was detected after caries removal, irrigation was performed with 3% sodium hypochlorite (NaOCl) solution, and a sterile cotton pellet soaked in 3% NaOCl was gently pressed over the exposed pulp wound surface to achieve hemostasis. The maximum diameter of the pulp exposure was measured clinically using a sterile periodontal probe under microscopic magnification and recorded in millimeters. The location of the pulp exposure hole, as well as the bleeding time were recorded. If hemostasis was achieved within 5 min, DPC can be performed. However, if hemostasis was not achieved, the affected tooth was excluded from the study scope, and instead, pulpotomy or root canal therapy was carried out.

Following successful hemostasis, the cavity was dried with a sterile cotton pellet. Based on the patient's randomized group allocation, one of the following three pulp capping agents was applied: Calxyl (CH paste, OCO Präparate GmbH, Dirmstein, Germany), ProRoot MTA (Dentsply Sirona, Johnson City, USA), or iRoot BP Plus (Innovative Bioceramix, Vancouver, Canada). The selected material was applied to cover the exposed pulp and surrounding dentin with a minimum thickness of 1.5 mm, ensuring complete adaptation without voids. A layer of light‐curing glass ionomer (Vitrebond, 3M Dental Products, USA) was then placed over the pulp‐capping agent, followed by a temporary seal with chemically cured glass ionomer cement (Fuji II Glass Ionomer Restorative, Self‐Cure, 1:1 Package, GC, Tokyo, Japan).

### 2.4 Follow‐Up and Outcomes

All the participants were then informed of postoperative instructions and scheduled an appointment for the subsequent follow‐up. 1 week after the intervention, a telephone follow‐up was conducted by a single instructor to record postoperative pain on the visual analogue scale (VAS) between 0 and 10 cm (Downie et al. 1978). One month after the intervention, patients were asked about any signs and symptoms, and clinically verified by percussion and pulp vitality test (cold test and EPT). In the absence of any symptoms, the temporary restoration was removed and changed to a permanent restoration under rubber dam isolation. All participants were advised to report any discomfort at any time during their follow‐up period. If the affected teeth remained asymptomatic, regular postoperative follow‐ups were scheduled at 3‐, 6‐, 12‐, and 24‐month intervals. During the follow‐up visits, the affected tooth underwent clinical examinations, including palpation, percussion, cold test, EPT, and radiographic evaluations of PAI. All these results were recorded on case report forms (CRF).

The primary outcome measurement was success rates among 3 groups at the 12‐ month follow‐up. Outcomes meeting both clinical and radiographic criteria were defined as successful. The clinical success criteria included the absence of spontaneous pain or tenderness to percussion in the affected tooth, no swelling, sinus tract, or tenderness in the soft tissues around the tooth, and responsiveness to the pulp vitality test. No pathosis evident such as periapical lesions or internal root resorption or external root resorption on radiographs was considered as radiographic success. Cases that failed to meet either of the criteria will be considered as failure. The radiographs were evaluated by 2 independent examiners blinded to the study aim.

The success rates at 3‐ and 6‐month follow‐up were secondary outcome measurements.

### 2.5 Statistical Analysis

Primary and secondary outcomes were compared among the three treatment groups using Chi‐square tests or Fisher's exact tests, as appropriate, based on expected cell counts. Baseline demographic and clinical characteristics were evaluated as potential prognostic factors, including age (≤ 40 vs. > 40 years), sex, arch, tooth position, lesion location, exposure site, exposure size, cold test response, and bleeding time. These variables were compared both across treatment groups at baseline to confirm comparability, and between successful and failed cases at the 3‐, 6‐, and 12‐month follow‐ups to determine associations with treatment outcomes.

In addition to the prespecified outcomes, an exploratory post hoc time‐to‐event analysis was performed using Kaplan–Meier survival curves to evaluate cumulative success probability over time. Log‐rank tests were used to compare survival distributions across treatment groups, as well as across different levels of each prognostic factor.

Analyses were conducted on a per‐protocol basis, including participants who completed the respective follow‐up; participants lost to follow‐up were treated as censored observations in time‐to‐event analyses. All data management, visualization, and statistical analyses were performed in R version 4.5.0 (R Core Team 2025) using the *tidyverse* package (Wickham et al. 2019). Statistical significance was set at a two‐sided *α* level of 0.05.

## 3 Results

**Figure 1:**
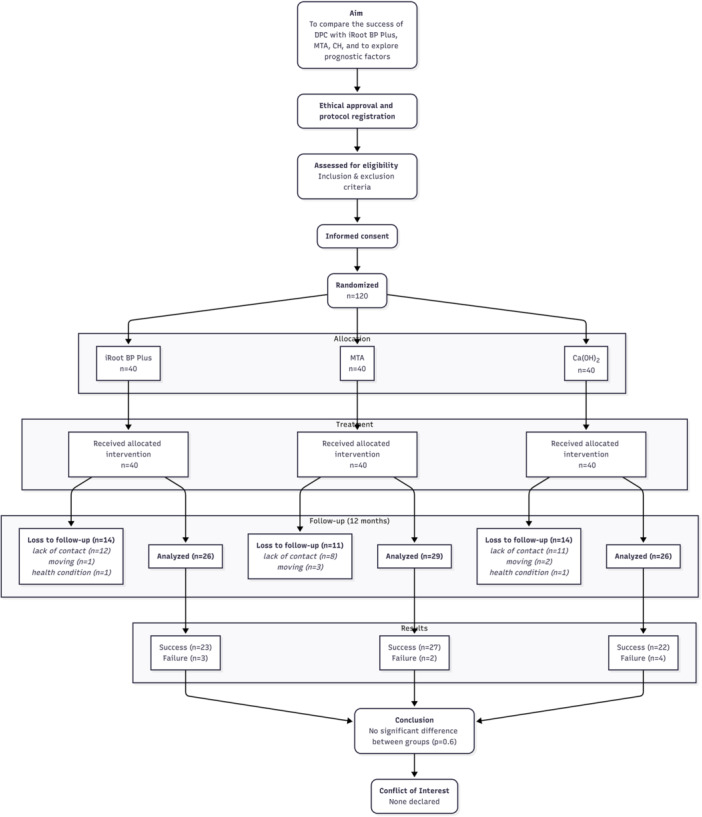
CONSORT flow diagram of participant enrollment and allocation. A total of 120 patients were screened for eligibility and randomly assigned into three groups (Calcium Hydroxide, MTA, and iRoot BP Plus). Follow‐up was conducted at 3, 6, and 12 months. The diagram shows participant flow, follow‐up completion, and analysis numbers for each group.

A total of 120 patients were randomized equally into the three treatment groups: iRoot BP Plus (*n* = 40), MTA (*n* = 40), and calcium hydroxide (*n* = 40) (Figure 1). Baseline demographic and clinical characteristics were comparable across groups (Table 1). Age distribution did not differ significantly (*p* > 0.9), with 40%–43% of participants aged ≤ 40 years. Female patients represented 58%–65% of each group (*p* = 0.8). The distribution of treated teeth (maxillary vs. mandibular, anterior vs. posterior) and lesion locations was also balanced across groups (*p* > 0.3) (Table 1).

**Table 1:** Baseline characteristics of patients by treatment group.

	Treatment group			
Characteristic	iRoot BP Plus *N* = 40^a^	MTA *N* = 40^a^	Calcium hydroxide *N* = 40^a^	*p* value
Age				> 0.9^b^
≤ 40 years	16 (43%)	16 (40%)	16 (40%)	
> 40 years	21 (57%)	24 (60%)	24 (60%)	
Sex				0.8^b^
Female	23 (61%)	23 (58%)	26 (65%)	
Male	15 (39%)	17 (43%)	14 (35%)	
Dentition				0.3^b^
Mandibular	17 (43%)	19 (48%)	24 (60%)	
Maxillary	23 (58%)	21 (53%)	16 (40%)	
Tooth position				> 0.9^c^
Posterior	36 (90%)	36 (90%)	37 (93%)	
Anterior	4 (10%)	4 (10%)	3 (7.5%)	
Lesion location				> 0.9^b^
Proximal surface only	13 (33%)	13 (33%)	13 (33%)	
Other	27 (68%)	27 (68%)	27 (68%)	
Exposure site location				0.9^b^
Other	20 (50%)	22 (55%)	22 (55%)	
Chamber wall	20 (50%)	18 (45%)	18 (45%)	
Exposure site size				0.3^c^
≤ 1 mm	37 (93%)	33 (83%)	37 (93%)	
> 1 mm	3 (7.5%)	7 (18%)	3 (7.5%)	
Cold test				0.6^b^
Responsive	28 (72%)	27 (71%)	32 (80%)	
Exaggerated	11 (28%)	11 (29%)	8 (20%)	
Bleeding time				0.003c,, d
≤ 1 min	40 (100%)	34 (85%)	40 (100%)	
> 1 min	0 (0%)	6 (15%)	0 (0%)	
Postoperative pain				0.5^b^
No	28 (72%)	33 (83%)	30 (79%)	
Yes	11 (28%)	7 (18%)	8 (21%)	

^a^ *n* (column %).

^b^ Pearson's Chi‐squared test.

^c^ Fisher's exact test.

d Significant at *p* < 0.01.

Most exposure sites were ≤ 1 mm (89%), and approximately half were located at the pulp chamber wall. Bleeding time at exposure, a clinical indicator of pulp inflammatory status rather than a material‐dependent variable, differed in its distribution among groups (*p* = 0.003). Hemostasis within 1 min was achieved in all iRoot BP Plus and calcium hydroxide cases, whereas 15% of teeth allocated to the MTA group required more than 1 min to achieve hemostasis (Table 1). Postoperative pain at 7 days did not differ significantly among the three treatment groups (*p* = 0.5) (Table 1).

This report reflects data collected up to the cutoff date of November 30, 2025, with a median follow‐up of 364 days. Over this period, 9 failure events were observed before censoring. The overall success rates at 3, 6, 12, and 24 months were 96.0%, 93.2%, 88.9%, and 72.7%, respectively (Table 2 and Table S1). No adverse or unexpected events were observed throughout the follow‐up. Representative clinical and radiographic documentation of successful and failed cases is provided in Figure S2.

**Table 2:** Association of treatment groups and baseline factors with direct pulp capping outcomes at 3, 6, and 12 months.

	3 Months			6 Months			12 Months		
Variables	Success *N* = 96^a^	Failure N = 4^a^	*p* value^b^	Success *N* = 82^a^	Failure *N* = 6^a^	*p*‐value^b^	Success *N* = 72^a^	Failure *N* = 9^a^	*p* value^b^
Treatment group			0.8			> 0.9			0.6
iRoot BP Plus	33 (97%)	1 (2.9%)		27 (93%)	2 (6.9%)		23 (88%)	3 (12%)	
MTA	32 (97%)	1 (3.0%)		28 (93%)	2 (6.7%)		27 (93%)	2 (6.9%)	
Calcium hydroxide	31 (94%)	2 (6.1%)		27 (93%)	2 (6.9%)		22 (85%)	4 (15%)	
Age			0.6			> 0.9			0.7
≤ 40 years	36 (95%)	2 (5.3%)		31 (94%)	2 (6.1%)		26 (87%)	4 (13%)	
> 40 years	59 (97%)	2 (3.3%)		50 (93%)	4 (7.4%)		45 (90%)	5 (10%)	
Sex			0.3			0.2			0.5
Female	60 (98%)	1 (1.6%)		51 (96%)	2 (3.8%)		45 (92%)	4 (8.2%)	
Male	36 (92%)	3 (7.7%)		31 (89%)	4 (11%)		27 (84%)	5 (16%)	
Dentition			0.3			> 0.9			> 0.9
Mandibular	45 (94%)	3 (6.3%)		41 (93%)	3 (6.8%)		36 (90%)	4 (10%)	
Maxillary	51 (98%)	1 (1.9%)		41 (93%)	3 (6.8%)		36 (88%)	5 (12%)	
Tooth position			0.049^c^			0.018^c^			0.077
Posterior	88 (98%)	2 (2.2%)		75 (96%)	3 (3.8%)		65 (92%)	6 (8.5%)	
Anterior	8 (80%)	2 (20%)		7 (70%)	3 (30%)		7 (70%)	3 (30%)	
Lesion location			0.012^c^			0.002^d^			0.001^d^
Proximal surface only	30 (88%)	4 (12%)		26 (81%)	6 (19%)		22 (73%)	8 (27%)	
Other	66 (100%)	0 (0%)		56 (100%)	0 (0%)		50 (98%)	1 (2.0%)	
Exposure site location			> 0.9			0.4			0.3
Other	50 (96%)	2 (3.8%)		43 (96%)	2 (4.4%)		39 (93%)	3 (7.1%)	
Chamber wall	46 (96%)	2 (4.2%)		39 (91%)	4 (9.3%)		33 (85%)	6 (15%)	
Exposure site size			0.059			0.024^c^			0.10
≤ 1 mm	87 (98%)	2 (2.2%)		74 (96%)	3 (3.9%)		64 (91%)	6 (8.6%)	
> 1 mm	9 (82%)	2 (18%)		8 (73%)	3 (27%)		8 (73%)	3 (27%)	
Cold test			0.6			0.3			> 0.9
Responsive	71 (95%)	4 (5.3%)		60 (91%)	6 (9.1%)		53 (88%)	7 (12%)	
Exaggerated	22 (100%)	0 (0%)		19 (100%)	0 (0%)		17 (89%)	2 (11%)	
Bleeding time			> 0.9			0.2			0.3
≤ 1 min	92 (96%)	4 (4.2%)		80 (94%)	5 (5.9%)		70 (90%)	8 (10%)	
> 1 min	4 (100%)	0 (0%)		2 (67%)	1 (33%)		2 (67%)	1 (33%)	

^a^ *n* (row %).

^b^ Fisher's exact test.

c Significant at *p* < 0.05.

d Significant at *p* < 0.01.

At 12 months, 81 of the 120 patients who received treatment were analyzed (Figure 1). Direct pulp capping demonstrated high overall success: iRoot BP Plus achieved an 88.5% success rate (23/26), MTA 93.1% (27/29), and calcium hydroxide 84.6% (22/26) (Table 2). Differences between materials were not statistically significant (*p* = 0.6). When treatment outcomes at 12 months were stratified by clinical and demographic factors, most variables—including age, sex, tooth location, exposure site, and bleeding time—were not significantly associated with success (Table 2). However, lesion location was strongly predictive (OR = 18.18, *p* = 0.001), with failures disproportionately concentrated in cases confined to the proximal surface.

Early follow‐up findings at 3 and 6 months were consistent with the 12‐month results, showing no significant differences between treatment groups (3‐month: *p* = 0.8, 6‐month: *p* > 0.9). At 6 months, all 6 failures occurred in cases with lesions confined to the proximal surface (*p* = 0.002). Size of exposure site > 1 mm had a markedly higher risk of failure (OR = 9.2, *p* = 0.024), and anterior teeth were more likely to fail compared with posterior teeth (OR = 10.7, *p* = 0.018) (Table 2). We also identify anterior teeth (*p* = 0.049) and lesion only on proximal surface (*p* = 0.012) as potential risk factors at 3 months.

**Figure 2:**
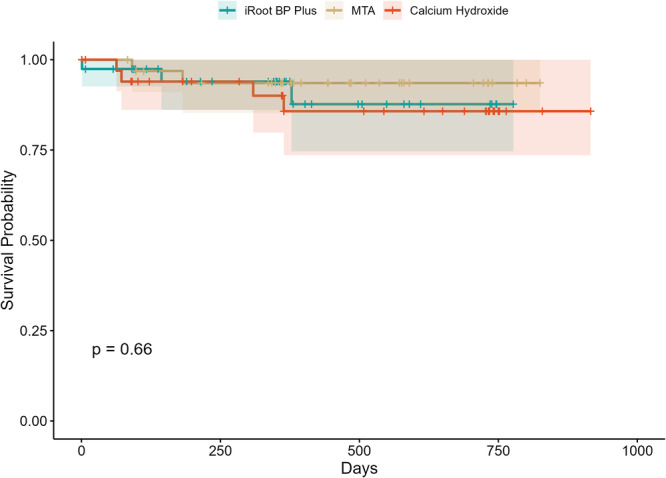
Kaplan–Meier survival curves for direct pulp capping success among the three treatment groups. Cumulative survival probabilities are shown for iRoot BP Plus, MTA, and calcium hydroxide. No significant differences were observed among groups (log‐rank *p* = 0.66). Shaded areas represent 95% confidence intervals.

Survival analysis with Kaplan–Meier estimates also showed high survival probabilities across all three capping materials, with the estimated overall survival probability of 89.3% (95% CI: 82.8%–96.4%). The estimated cumulative survival probability was 87.7% for iRoot BP Plus (95% CI: 74.6%–100%), 93.5% for MTA (95% CI: 85.3%–100%), and 85.7% for calcium hydroxide (95% CI: 73.5%–100%). Survival curves did not differ significantly among the groups (log‐rank test, *p* = 0.66) (Figure 2). However, Kaplan–Meier curves with log‐rank testing supported the mentioned several significant risk factors for failure: lesions limited to the proximal surface (*p* = 0.0003), exposure site size > 1 mm (*p* = 0.015) and involvement of anterior teeth (*p* = 0.013) (Figure S2).

## 4 Discussion

In clinical practice, root canal therapy (RCT) is often performed when pulp exposure is detected during or after caries removal, given the difficulty of determining the extent of pulpal inflammation. However, the loss of vital pulp function after RCT—formation, nutrition, defense, and sensation—reduces tooth resilience and increases the risk of fracture, often compromising long‐term survival compared with teeth retaining vital pulp (Caplan et al. 2005; Xie et al. 2021). VPT, including DPC, provides a biologically based alternative that preserves pulp vitality and has been shown to improve long‐term tooth retention compared with RCT (Lin et al. 2020). Historically, DPC was considered predictable only for traumatic exposures or immature teeth, as calcium hydroxide yielded poor outcomes in caries‐related exposures, with success rates as low as 37% at 5 years and 13% at 10 years (Barthel et al. 2000). More recently, advances in biomaterials, aseptic technique, and magnification have markedly improved DPC outcomes. A systematic review reported an overall DPC success rate of 83% in permanent teeth over the past two decades (Prasertsuksom et al. 2024) supporting its role as an alternative to RCT in selected mature teeth.

In this study, the overall 12‐month success rate of DPC was 88.9%. Success did not differ significantly among materials: iRoot BP Plus (88.5%), MTA (93.1%), and CH (84.6%). Kaplan–Meier analysis showed similar cumulative survival probabilities, with no significant difference among groups. Our findings are consistent with previous clinical studies. Brizuela et al. reported no differences among MTA, CH, and Biodentine in young permanent teeth at 12 months, with success rates closely matching ours (Brizuela et al. 2017). Similarly, a retrospective study found no significant difference between MTA and CH at 24 months (93% vs. 90%) (Çalışkan and Güneri 2017). Other investigations, however, highlight potential advantages of MTA. Kundzina et al. (2017) reported higher 3‐year survival with MTA (85%) compared to CH (52%), estimating that one root canal treatment could be avoided for every three cases treated with MTA. This difference has been attributed to the cytotoxicity and poor sealing of some CH formulations (e.g., Dycal), while MTA promoted more favorable biological responses (Manaspon et al. 2021). Another clinical trial in mature molars also reported significantly higher 12‐month success with MTA (93%) than with CH (69%), suggesting that baseline pulpal status and material properties both influence outcomes (Suhag et al. 2019).

The overall internal validity of the trial was strong, and the clinical context of the cases supports good applicability of the findings to routine practice. In our study, the high 12‐month success rate of iRoot BP Plus was comparable to previous findings. Chen et al. (2023) observed long‐term survival rates of 92% at 1 year and 72% at 5 years in a large cohort of 354 teeth, with an overall success rate of 85%. Experimental studies further indicate that iRoot BP Plus shares similar biological properties with MTA, including promotion of calcific bridge formation, favorable pulp responses, and dentin mineralization, while in some models it demonstrated superior induction of tertiary dentin (Zhu et al. 2014; Shi et al. 2016; Okamoto et al. 2018). Collectively, these findings suggest that iRoot BP Plus is an effective capping material with performance comparable to MTA, although additional long‐term clinical evidence in mature permanent teeth is still needed.

Beyond material choice, several clinical risk factors were significantly associated with outcome. Teeth with caries confined to the proximal surface showed higher failure rates at 3, 6, and 12 months, consistent with earlier studies that reported reduced survival in proximal lesions (Marques et al. 2015) and in VPT procedures more broadly (Coll et al. 2013; Wassel et al. 2023). The lower success in proximal lesions likely reflects greater challenges in achieving isolation, complete caries removal, and durable restoration, as well as higher bacterial load and thinner residual dentin. Restoration quality in proximal sites also tends to be inferior, increasing the risk of microleakage, food impaction, and fracture, all of which compromise pulp healing. Given this strong association, future trials should consider stratified randomization by lesion location to better control for confounding.

Anterior teeth also showed higher failure rates than posterior teeth, particularly at 3 and 6 months, echoing prior reports (Horsted et al. 1985). This may relate to anatomical and biomechanical differences: anterior teeth typically have a single canal with rapid pulp infection spread, are exposed to greater horizontal stress (Soares et al. 2012), and are more prone to restoration failure, all of which may reduce DPC success. Posterior teeth, with multiple canals, may show false‐positive pulp vitality responses if some canals remain vital, partly explaining their higher observed success. Notably, anterior teeth in our cohort were significantly more likely to exhibit lesions confined to the proximal surface (OR = 11.85), indicating a strong correlation between these two variables. As a result, their individual contributions to failure risk cannot be interpreted independently.

Pulp exposure size was another factor. Although evidence is mixed, we observed lower success at 6 months when exposures exceeded 1 mm, consistent with reports linking larger exposures to more extensive pulpal injury (Ricucci et al. 2023). However, this association was not significant at 3 or 12 months, suggesting other confounding factors may influence long‐term outcomes.

Hemostasis duration has been widely regarded as a prognostic indicator of pulp status. Prolonged bleeding suggests deeper inflammation and poorer prognosis (Matsuo et al. 1996; Suhag et al. 2019). In our study, patients requiring > 5 min for hemostasis were excluded, and standardized hemorrhage control with 3% NaOCl under magnification was used in all cases, following AAE recommendations. NaOCl not only aids hemostasis but also removes clot and bacterial contaminants without impairing pulp healing (Hafez et al. 2002). Recent clinical trials data further support its role in reducing postoperative pain and improving DPC outcomes (Ballal et al. 2024).

This study has limitations. The 32% dropout rate exceeded the 20% anticipated in our sample size calculation. Much of this attrition occurred during periods when routine dental services were suspended or restricted due to COVID‐19, making scheduled follow‐ups difficult for many patients (De Souza et al. 2023). Although baseline characteristics remained balanced between retained and lost participants, the reduced sample size limited statistical power to detect smaller between‐group differences. Given that patients typically return when symptoms persist, those lost to follow‐up were likely to have remained asymptomatic, suggesting our success rates may be conservative estimates. Additionally, long‐term follow‐up beyond 12 months was unavailable for most participants, limiting evaluation of material stability and delayed complications. The reduced final cohort also precluded multivariable modeling of risk factors, leaving the possibility of residual confounding. Future studies with larger samples and improved follow‐up strategies are needed to confirm these results.

## 5 Conclusions

Overall, this study supports direct pulp capping as a reliable and biologically sound alternative to root canal therapy in carefully selected mature permanent teeth. Short‐ to medium‐term outcomes were favorable across all three materials. Clinical factors such as proximal caries, anterior tooth location, and larger exposure size were identified as significant risk factors for failure, underscoring the importance of case selection and meticulous technique. Despite limitations related to sample attrition, these findings reinforce the growing evidence that vital pulp therapy can extend tooth longevity while preserving pulp vitality, and further long‐term, large‐scale studies are warranted to confirm these results.

## Author Contributions

**Yutong Li:** conceptualization, data curation, formal analysis, methodology, project administration, validation, writing – original draft. **Kangrui Zeng:** conceptualization, formal analysis, funding acquisition, investigation, methodology. **Boshu Chen:** formal analysis, software, validation, visualization, writing – original draft. **Qiongyi Kang, Lili Huang, Hong Huang, Tingting Li, Yueping Pan, Shujun Ran:** data curation, formal analysis, investigation. **Wenwei Xia:** conceptualization, funding acquisition, project administration, supervision, writing – review and editing.

## Conflicts of Interest

The authors declare no conflicts of interest.

## Supporting information


**Figure S1:** Representative clinical and radiographic outcomes following direct pulp capping. (A) Long‐term success (tooth 46). (A1) Preoperative view showing deep caries with no periapical radiolucency. (A2–A4) Follow‐up at 3, 12, and 24 months demonstrating maintained vitality and absence of periapical pathology. (B) Success with sequential follow‐up (tooth 15). (B1) Preoperative view. (B2–B5) Follow‐up at 3, 6, 12, and 24 months showing stable periapical status. (C) Failure (tooth 16). 12‐month follow‐up demonstrating loss of vitality and development of periapical radiolucency. (D) Clinical procedure under magnification (tooth 25). (D1) Caries removal under rubber dam isolation. (D2) Disinfection with 3% sodium hypochlorite (NaOCl). (D3) Application of pulp‐capping material (~1.5 mm thickness).

**Figure S2:** Kaplan‐Meier survival curves for direct pulp capping success among different levels of prognostic factors. Cumulative survival probabilities are shown for different prognostic factors. (A) Lesion confined to proximal surface or not. (B) Exposure site size > 1 mm or not. (C) Anterior or posterior teeth. Shaded areas represent 95% confidence intervals.

**Table S1:** Association of Treatment Groups and Baseline Factors with Direct Pulp Capping Outcomes at 24 Months.

Supporting File

## Data Availability

The data that support the findings of this study are available on request from the corresponding author. The data are not publicly available due to privacy or ethical restrictions.
