# Supplementary material for: Clinical Evaluation of Three Direct Pulp Capping Materials in Caries‐Induced Pulpitis of Mature Permanent Teeth: A Randomized Controlled Trial
Source: Clin Exp Dent Res. 2026 May 26;12(3):e70367. doi: 10.1002/cre2.70367 (PMC13239863; doi:10.1002/cre2.70367)
Supplement: Supplementary file 1 — Figure S1: Representative clinical and radiographic outcomes following direct pulp capping. (A) Long‐term success (tooth 46). (A1) Preoperative view showing deep caries with no periapical radiolucency. (A2–A4) Follow‐up at 3, 12, and 24 months demonstrating maintained vitality and absence of periapical pathology. (B) Success with sequential follow‐up (tooth 15). (B1) Preoperative view. (B2–B5) Follow‐up at 3, 6, 12, and 24 months showing stable periapical status. (C) Failure (tooth 16). 12‐month follow‐up demonstrating loss of vitality and development of periapical radiolucency. (D) Clinical procedure under magnification (tooth 25). (D1) Caries removal under rubber dam isolation. (D2) Disinfection with 3% sodium hypochlorite (NaOCl). (D3) Application of pulp‐capping material (~1.5 mm thickness). [file CRE2-12-e70367-s001.pdf]

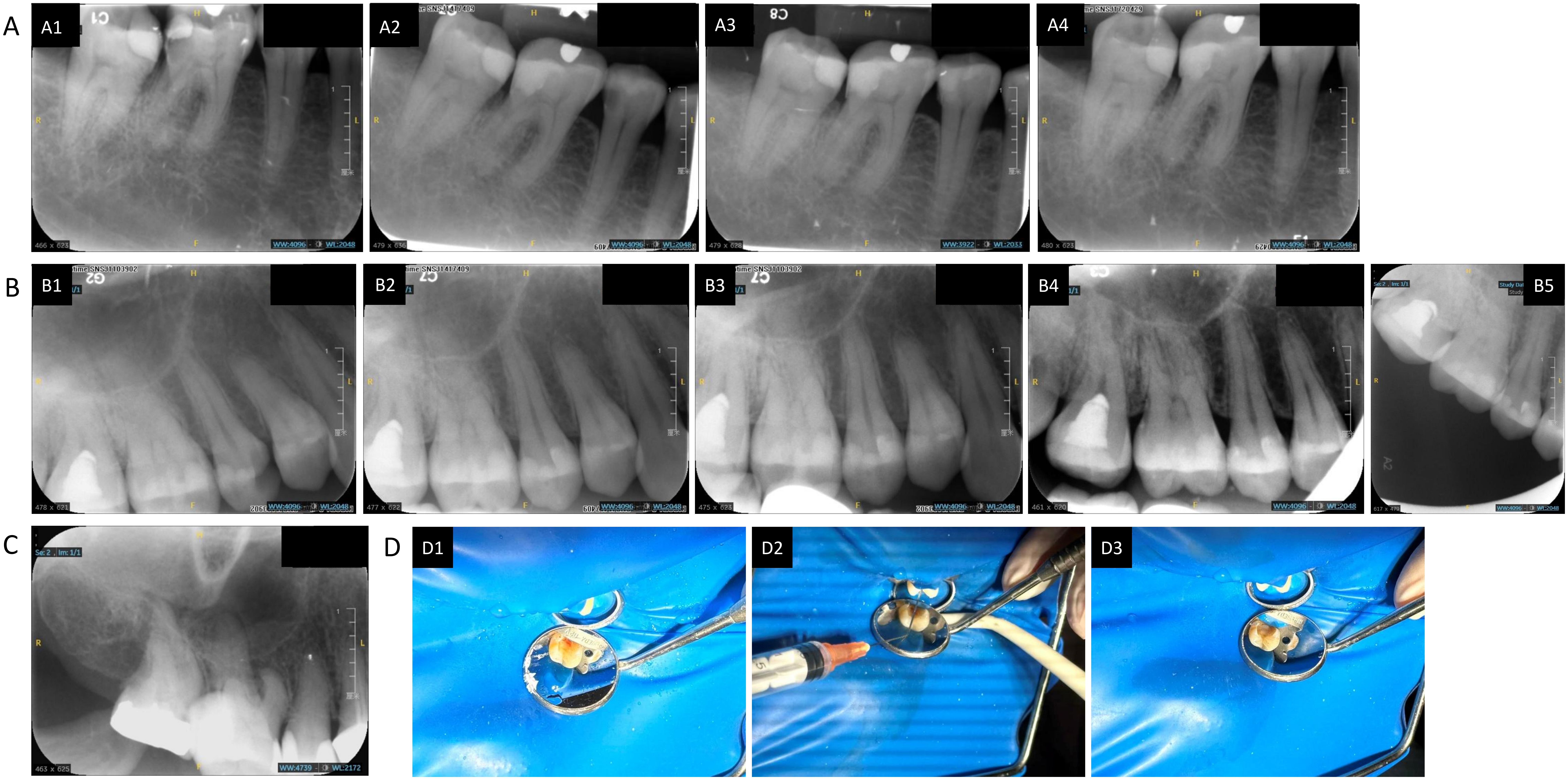

Figure S1. Representative clinical and radiographic outcomes following direct pulp capping.
(A) Long-term success (tooth 46). (A1) Preoperative view showing deep caries with no periapical radiolucency. (A2–A4) Follow-up at 3, 12, and 24 months demonstrating maintained vitality and absence of periapical pathology. (B) Success with sequential follow-up (tooth 15). (B1) Preoperative view. (B2–B5) Follow-up at 3, 6, 12, and 24 months showing stable periapical status. (C) Failure (tooth 16). 12-month follow-up demonstrating loss of vitality and development of periapical radiolucency. (D) Clinical procedure under magnification (tooth 25). (D1) Caries removal under rubber dam isolation. (D2) Disinfection with 3% sodium hypochlorite (NaOCl). (D3) Application of pulp-capping material (~1.5 mm thickness).
