# Supplementary figures and images for: Clinical Evaluation of Three Direct Pulp Capping Materials in Caries‐Induced Pulpitis of Mature Permanent Teeth: A Randomized Controlled Trial
Source: Clin Exp Dent Res. 2026 May 26;12(3):e70367. doi: 10.1002/cre2.70367 (PMC13239863; doi:10.1002/cre2.70367)

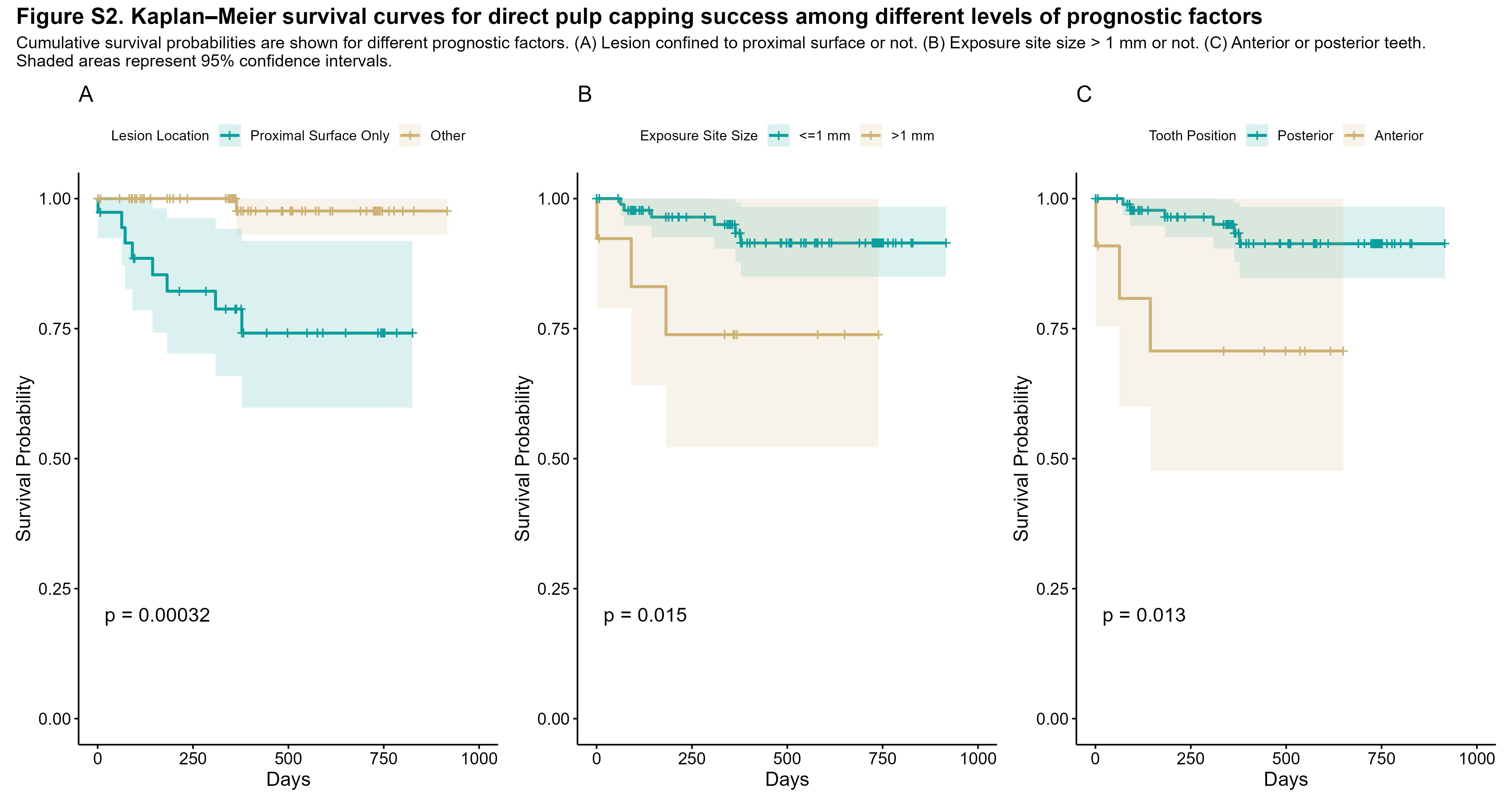

Supplement: Supplementary file 2 — Figure S2: Kaplan‐Meier survival curves for direct pulp capping success among different levels of prognostic factors. Cumulative survival probabilities are shown for different prognostic factors. (A) Lesion confined to proximal surface or not. (B) Exposure site size > 1 mm or not. (C) Anterior or posterior teeth. Shaded areas represent 95% confidence intervals. [file CRE2-12-e70367-s004.png]
