# Supplementary material for: Clinical Evaluation of Three Direct Pulp Capping Materials in Caries‐Induced Pulpitis of Mature Permanent Teeth: A Randomized Controlled Trial
Source: Clin Exp Dent Res. 2026 May 26;12(3):e70367. doi: 10.1002/cre2.70367 (PMC13239863; doi:10.1002/cre2.70367)
Supplement: Supplementary file 3 — Table S1: Association of Treatment Groups and Baseline Factors with Direct Pulp Capping Outcomes at 24 Months. [file CRE2-12-e70367-s002.docx]

|  | **24 Months** | |  |
| --- | --- | --- | --- |
| **Variables** | **Success** N = 24*^†^* | **Failure** N = 9*^†^* | **p-value***^‡^* |
| **Treatment Group** |  |  | 0.8 |
| iRoot BP Plus | 5 (63%) | 3 (38%) |  |
| MTA | 8 (80%) | 2 (20%) |  |
| Calcium Hydroxide | 11 (73%) | 4 (27%) |  |
| **Age** |  |  | 0.7 |
| ≤40 years | 8 (67%) | 4 (33%) |  |
| >40 years | 16 (76%) | 5 (24%) |  |
| **Sex** |  |  | 0.4 |
| Female | 16 (80%) | 4 (20%) |  |
| Male | 8 (62%) | 5 (38%) |  |
| **Dentition** |  |  | >0.9 |
| Mandibular | 12 (75%) | 4 (25%) |  |
| Maxillary | 12 (71%) | 5 (29%) |  |
| **Tooth Position** |  |  | 0.015* |
| Posterior | 24 (80%) | 6 (20%) |  |
| Anterior | 0 (0%) | 3 (100%) |  |
| **Lesion Location** |  |  | 0.004** |
| Proximal Surface Only | 7 (47%) | 8 (53%) |  |
| Other | 17 (94%) | 1 (5.6%) |  |
| **Exposure Site Location** |  |  | 0.11 |
| Other | 17 (85%) | 3 (15%) |  |
| Chamber Wall | 7 (54%) | 6 (46%) |  |
| **Exposure Site Size** |  |  | 0.052 |
| ≤1 mm | 23 (79%) | 6 (21%) |  |
| >1 mm | 1 (25%) | 3 (75%) |  |
| **Cold Test** |  |  | 0.7 |
| Responsive | 14 (67%) | 7 (33%) |  |
| Exaggerated | 8 (80%) | 2 (20%) |  |
| **Bleeding Time** |  |  | 0.3 |
| ≤1 min | 24 (75%) | 8 (25%) |  |
| >1 min | 0 (0%) | 1 (100%) |  |
| *^†^*n (Row %) | | | |
| *^‡^*Fisher's exact test | | | |
| *significant at p < 0.01 | | | |
| **significant at p < 0.01 | | | |

**Table S1. Association of Treatment Groups and Baseline Factors with Direct Pulp Capping Outcomes at 24 Months**
